# The Impact of Za’atar Antioxidant Compounds on the Gut Microbiota and Gastrointestinal Disorders: Insights for Future Clinical Applications

**DOI:** 10.3390/antiox12020426

**Published:** 2023-02-09

**Authors:** Mohamad Khalil, Hala Abdallah, Danute Razuka-Ebela, Maria Calasso, Maria De Angelis, Piero Portincasa

**Affiliations:** 1 Clinica Medica “A. Murri”, Department of Biomedical Sciences & Human Oncology, University of Bari Aldo Moro, 70121 Bari, Italy; 2 Department of Soil, Plant and Food Sciences, University of Bari Aldo Moro, via Amendola 165/a, 70126 Bari, Italy; 3 Institute of Clinical and Preventive Medicine, University of Latvia, 1586 Riga, Latvia

**Keywords:** Za’atar, microbiota, gastrointestinal diseases, polyphenols, antioxidants

## Abstract

Since the gut microbiota plays a pivotal role in host homeostasis and energy balance, changes in its composition can be associated with disease states through the promotion of immune-mediated inflammatory disorders and increasing intestinal permeability, ultimately leading to the impairment of intestinal barrier function. Za’atar is one of the most popular plant-based foods in the Eastern Mediterranean region. Za’atar is a mixture of different plant leaves, fruits, and seeds and contains hundreds of antioxidant compounds, especially polyphenols, and fiber, with pre-clinical and clinical evidence suggesting health-promoting effects in cardiovascular and metabolic disease. Za’atar compounds have also been studied from a gastrointestinal perspective, concerning both gut microbiota and gastrointestinal diseases. Antioxidants such as Za’atar polyphenols may provide beneficial effects in the complex interplay between the diet, gut microbiota, and intestinal permeability. To our knowledge, no studies have reported the effects of the whole Za’atar mixture, however, based on the pre-clinical studies published on components and single compounds found in Za’atar, we provide a clinical overview of the possible effects on the gastrointestinal tract, focusing mainly on carvacrol, rosmarinic acid, gallic acid, and other polyphenols. We also cover the potential clinical applications of Za’atar mixture as a possible nutraceutical in disorders involving the gastrointestinal tract.

## 1. Introduction

The gastrointestinal tract is considered the largest surface of the human body exposed to the external environment [1]. The gut barrier has several essential functions, especially the absorption of nutrients and defense against harmful macromolecules [2]. The gut barrier is a complex multilayer system consisting of both an external “physical” barrier and an inner “functional” immune barrier. The physiological “healthy” interaction of both barriers ensures the selective permeability of nutrients, water, and bacterial products. Different factors can alter the function, physiology, and homeostasis of the gastrointestinal tract potentially leading to the development of a wide range of functional and inflammatory gastrointestinal disorders. Furthermore, the long-term effects of stress could affect gut-liver and gut-brain interactions. Stress-related factors include altered gastrointestinal motility and increased intestinal permeability with negative effects on the diversity of gut microbiota [3]. A close relationship exists between nutrients (dietary fiber, protein, fat), gut microbiota and its products (e.g., short-chain fatty acids, lipopolysaccharides), and the intestinal barrier in both health and disease [1]. Although enormous effort has been put into studies to investigate the role of dietary intervention in improving intestinal barrier function and preventing increased intestinal permeability in animals, the relevance of these results for human health is still poorly understood. Za’atar mixture is the most popular plant-based food in the Eastern Mediterranean area. The original traditional Lebanese mixture consists of various plants, fruits, and seeds. These plants contain a panel of biologically active compounds—such as polyphenolic compounds (PC), essential oils (EOs), and fiber—some of which have displayed potential health-promoting effects in cardiovascular disease and metabolic syndrome. The effects of the whole Za’atar mixture are still missing, however, studies on some components and compounds found in Za’atar on the gastrointestinal tract have also been studied regarding gut microbiota and intestinal permeability in both animals and humans. Herein we provide an overview of the intestinal barrier from a functional perspective, as well as in terms of intestinal permeability and its association with gut microbiota. In addition, we focused on the Za’atar mixture, described its general history and cultural aspects, and then we detailed the components, chemical/phytochemical composition, and nutritional profile. Finally, we implemented the review with all available data on the potential effects of Za’atar components on the gastrointestinal tract and gut microbiota including cellular, animal, and human studies. Since there are no studies describing the effects of the whole Za’atar mixture, here we focused on the effects of single components and/or single (poly)phenolic compounds to highlight to possible health-promoting effects of the mixture.

## 2. Gut Physiology and Microbiota

The microbial barrier comprises the first level of the gut barrier and consists of hundreds of trillions of resident microorganisms including bacteria, viruses, fungi, bacteriophages, and protists [4]. In the human gut, the two major phyla include *Firmicutes* (mainly gram-positive bacteria with facultative anaerobes, anaerobes, bacilli, and cocci) and *Bacteroidetes* (mainly gram-negative *Bacteroides*, *Alistipes*, *Parabacteroides*, and *Prevotella* spp.) [5,6]. The gut microbiota plays an essential role in the digestion of nutrient and non-nutrient molecules, vitamin synthesis and metabolism, and the biotransformation of intestinal primary bile acids (BAs) to secondary BAs, which play a key role in lipid digestion and hormone-like agonist signaling. In addition, the gut microbiota has a crucial impact on the induction and function of the human immune system by influencing the maturation of several immune cells in intestinal lymphoid tissue and mucosa. The gut microbiota produces physiologically active molecules such as short-chain fatty acids (SCFAs) that have direct effects on the gut barrier and distant organs including, but not limited to, the brain, liver, and heart [7]. In addition, gut microbiota modulate nutritionally derived metabolites such as tryptophan which exerts a protective effect against intestinal inflammation and bacterial overgrowth [8]. The gut microbiota converts tryptophan to serotonin, transforms food polyphenols to bioactive smaller polyphenols, and affects the availability of neurotransmitters [9,10].

**Figure 1:**
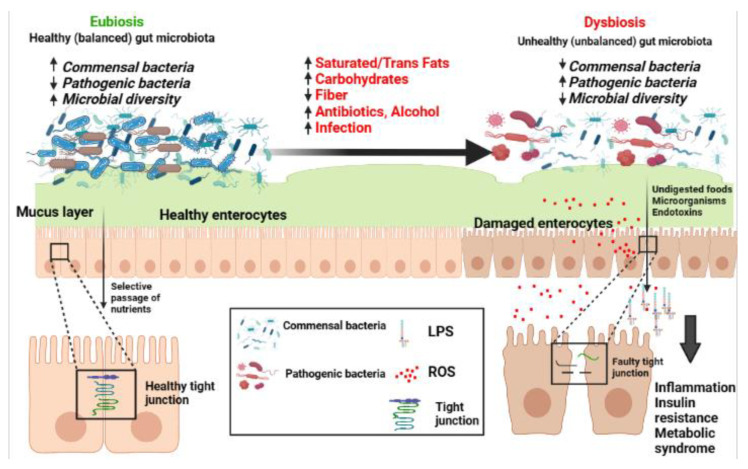
The transition from a balanced (Eubiosis) to an unbalanced (dysbiosis) gut microbiota and the different factors involved in gut dysbiosis. Abbreviations: LPS: lipopolysaccharide; ROS: reactive oxygen species; **↑** increased; ↓ decreased. The figure is developed using https://biorender.com/ (accessed on 12 December 2022).

The mucosal barrier is the first line of defense against external pathogens. It separates the external from the internal environment, protects against toxins and the passage of bacteria, and facilitates the transport of water, nutrients, ions, and solutes [11] (Figure 1). The intestinal mucosa plays the dual role of enforcing both defense and digestion by adapting to the colonization by commensal bacteria that participate in digestive processes and the induction of the intestinal immune system [12]. The epithelial intestinal barrier consists of a highly organized complex of intercellular apical tight junction proteins and tight junction-associated proteins including claudin, occludin, intracellular plaque zonula occludens (ZO) 1 and 2, cingulin, and other junctional adhesion proteins [13]. These tight junctions distribute cells into apical and basolateral regions. Despite being the first line of defense in the intestinal lumen, the epithelial barrier manages to simultaneously regulate the passive diffusion of macromolecules and solutes beneficial to the host and to prevent the passage of harmful pathogens [14] (Figure 1).

The dysbiosis of gut microbiota is defined as an altered microbial community with an increased population of pathogenic bacteria resulting in the increased release of bacterial endotoxins, especially lipopolysaccharides (LPS) that can alter the function of the intestinal barrier on a fundamental level by LPS-induced damage of enterocytes, leading to increased intestinal permeability. The intestine becomes “leaky,” allowing for the passage of LPS into the bloodstream, in turn activating inflammatory pathways resulting in systemic inflammation and potentially metabolic disorders [15,16,17,18].

## 3. Za’atar Mixture

Over the past ten years, interest in Za’atar has greatly increased within the scientific and industrial communities. Recent advances have been made in unraveling the multilevel properties of Za’atar plants or mixtures in terms of health-promoting effects, as well as cultivation, processing, production, and packing [19,20,21]. Za’atar is of economic and cultural importance in the Mediterranean region. More interestingly, it possesses nutraceutical and bio-functional properties as it is rich in (poly)phenolic compounds (PC), minerals, and fiber. So, it comes as no surprise, that there is both national and international interest in Za’atar [22,23,24]. 

**Figure 2:**
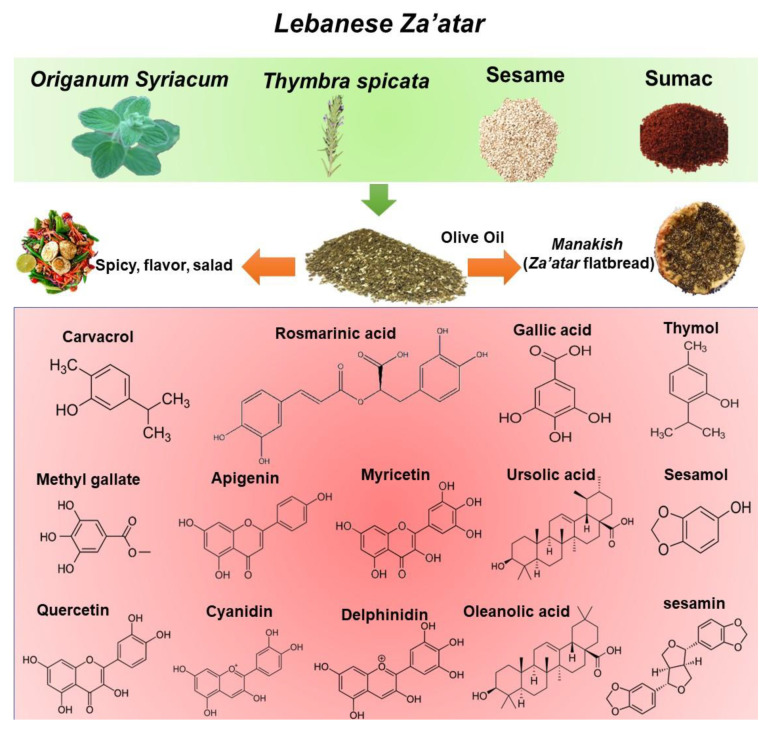
The composition of Za’atar mixture with polyphenols.

The term “Za’atar” is used to refer to both a type of plant as well as to a mixture of plants and spices [25]. Za’atar plants include *Origanum*, *Satureja*, *Thymbra*, and *Thymus* with high content of essential oil content, especially carvacrol and thymol [22,23,24]. As a mixture, the composition of Za’atar can vary by region and household, usually depending on the local availability of ingredients. Typically, in Lebanon, Za’atar mixture includes dried leaves of *Origanum syriacum* (also referred to as Za’atar) and *Thymbra spicata* (also known as wild Za’atar) mixed with *Rhus coriaria* ground fruit (sumac), toasted sesame seeds, and salt (Figure 2). Za’atar is commonly mixed with olive oil to form a spread for Lebanese flatbreads named “Mankoushe” [26]. The detailed components and polyphenolic composition and contents of the Za’atar mixture are shown in Figure 2.

In traditional books on Lebanese herbal medicine Za’atar (or Sa’atar) is presented as a special and distinct class of edible and medicinal herbs (*O. syriacum* and *T. spicata*) for the treatment of gastrointestinal diseases. Specifically, it is described that these herbs protect the intestinal mucosal barrier, reduce abdominal pain, and help prevent constipation by keeping the stool moist. These traditional books on herbal medicine also describe the gastroprotective and hepatoprotective effects of Za’atar plants [23]. Sumac, a component of the Za’atar mixture, is traditionally used in folk medicine for the treatment of chronic diarrhea, vomiting, and hemorrhoids. Water infusions of sumac could reduce inflammation, relax and protect the stomach, protect the liver, and help the bile reach the intestine [27].

### 3.1. Chemical and Phytochemical Composition of Mixed Za’atar

*O. syriacum* and *T. spicata* extracts contain high amounts of antioxidant compounds such as polyphenols including phenolic acids and flavonoids with a wide range of biological and pharmacological activities [28,29,30,31,32]. Rosmarinic acid (RA) is one of the main polyphenolic substances in both plants [24,33,34,35]. Ursolic and oleanolic acids are also present in high quantities in *O. syriacum* extracts [35]. Phenolic monoterpenes, especially carvacrol (CVL), are abundant in both essential oils and organic extracts, with the total essential oil content ranging from 60 to 70% [36,37]. One of the most abundant subclasses of polyphenols present in *O. syriacum* and *T. spicata* extracts are flavonoids—mostly luteolin and apigenin in aglycone or glucoside form [38,39] (Figure 2).

Sesame (*Sesamum indicum* L.) is an annual plant belonging to the *Pedaliaceae* family. Sesame seeds have been used for several thousands of years in Eastern, Mediterranean, and African cultures to flavor foods [40]. Nutritionally, sesame seeds are rich in oil (50–60%) and protein (18–25%) and contain carbohydrates (13.5%) and ash (5%) [41]. Sesame seeds contain high levels of unsaturated fatty acids—mainly oleic (43%), linoleic (35%), palmitic (11%), and stearic acids (7%)—collectively comprising 96% of the total fatty acid content [42]. Sesame seeds also contain protein, especially high levels of methionine, tocopherol, and phytosterol, as well as minerals and lignans, such as sesamol and sesamin—natural phenolic compounds and major lignans [43] (Table 1).

*Rhus coriaria* or sumac is rich in hydrolyzable tannins such as gallic acid, methyl gallate, and their derivatives, [44,45]. The most abundant organic acids found in *R. coraria* fruit are malic acid isomers and their derivatives. Sumac is abundant also in phenolic acids [46] and flavonoids including myricitrin, apigenin, quercetin, and kaempferol as aglycone or glycoside derivatives [46,47]. Cyanidin and delphinidin derivatives are the most abundant anthocyanin present in sumac and give its fruits their rich red color [44].

**Table 1:** The components of Za’atar mixture by name and useful contents.

Scientific Name	Local Name (Lebanon)	Used Part	Phenolic Contents mgGAE/g Dry Extract	Fiber g/100 g	Oils/Fatty Acids g/100 g	Essential Oils	Minerals (in Order of Abundance)	Vitamins	References
*Origanum Syriacum*	Za’atar (Zouba’a)	Leaves	120–250	15	0.13	Carvacrol, thymol	ND	C	[38,48,49,50]
*Thymbra spicata*	Wild Za’atar (Za’atar dakki)	Leaves	90–250	ND	PA OA	Carvacrol	ND	ND	[37,38,39]
*Rhus coriaria*	Sumac	Fruits	48–140	14	7–18 (37.7% OA, 34.8% LA, 27.4% PA, 17.3% SA)	β-caryophyllene, α-pinene	K, Ca, Mg, P	B1, B2, B6, B12, C	[51,52,53,54]
*Sesamum indicum*	Sesame	Seeds	7–80	6–10.8	51.9 OA (43%) LA (35%), PA (11%) SA (7%)	ND	Ca, K, P, Mg, Fe	Carotene, B1, B2, B3, E	[55,56,57]

**Abbreviations:** PA—palmitic acid; OA—oleaic acid; SA—stearic acid; LA—linoleic acid, K-Potasium; Ca-calcium; Mg-magnesium; Fe-iron.

### 3.2. Effects of Za’atar Compounds on Microbiota and Intestinal Disorders: Cellular and Animal Studies

Preclinical evidence including cellular and animal studies explored the potential effects of polyphenols on gut microbiota and gut diseases. These compounds interact bidirectionally with the gut microbiota and can modulate the composition by exerting prebiotic-like mechanisms and inhibiting pathogenic bacteria [58,59]. On the other hand,, after the intake of polyphenol-rich foods, the gut microbiota plays a fundamental role in enhancing biotransformation and bioavailability and, thus, the bio-activity of polyphenols. The effect of polyphenols and polyphenol-rich foods on microbial growth, in vitro, in vivo, and ex vivo is well documented [59]. Studies reported that polyphenols can inhibit the growth of several pathogens, including *E. coli*, *S. enteritidis*, *S. typhimurium* and *C. perfringens*, *L. monocytogenes*, *H. pylori*, *B. cereus*, *P. aeruginosa*, and *S. aureus* [60,61,62,63]. Polyphenols and their metabolites showed a prebiotic-like effect on shaping gut microbiota and modulation of inflammatory responses by enhancing the abundance of beneficial bacteria including *Lactobacillus*, *Bifidobacterium*, *Faecalibacterium prausnitzii*, and *Roseburia* [64,65,66,67]. In the following paragraphs we report the available studies about the effects of Za’atar compounds on microbiota and gastrointestinal disorders, all findings summarized in the Table 2. 

#### 3.2.1. *Origanum syriacum* and *Thymbra spicata* Extracts

*O. syriacum* and *T. spicata* are considered ancient herbal remedies are known as biblical hyssop [109]. *O. syriacum* showed protective effects in an animal peptic ulcer model with a significant decrease in ulcer number and size, similar to that of clinically approved lansoprazole in the prophylactic model [69]. The antiparasitic potential of *O. syriacum* essential oils (EOs) against the nematode *Anisakis simplex* has been demonstrated by Lopez et al. [68]. It must be noted that patients infected with *Anisakis simplex* developed allergic responses and increased intestinal permeability [110,111]. The EOs obtained from *O. syriacum* demonstrated potent antifungal activity against several species of fungi potentially involved in intestinal infection and disease [112]. Similarly to *O. syriacum*, several studies have evaluated the composition and antimicrobial and antiparasitic effects of EOs derived from *T. spicata* [37,113,114,115].

#### 3.2.2. Carvacrol, Thymol, and Essential Oils (EOs)

Over the past two decades, increasing evidence suggests that EOs derived from aromatic plants could exert a positive effect on gut microbiota and associated intestinal disorders. These oils exert selective antimicrobial effects against pathogenic bacteria, fungi, and parasites, and favorable effects on commensal and beneficial bacteria [116,117,118]. Supplementation with EOs rich in carvacrol and thymol in weaned piglets resulted in beneficial changes in gut microbiota composition demonstrated by an increase in the relative abundance of some beneficial species such as *Bacilli*, *Lactobacillales*, and *Veillonellaceae* [70]. Similarly, supplementation with oregano essential oil (OEO) rich in carvacrol and thymol resulted in changes in the gut microbiota accompanied by improved piglet health and performance [71]. OEO supplementation during lactation showed an increase in the relative abundance of *Lactobacillaceae*, as well as *Fibrobacteriaceae* and *Akkermansiaceae* involved in fiber digestion. At two and four weeks, a relative decrease in *Enterobacteriaceae* and an increase in butyrate-producing *Lachnospiraceae* was observed. The authors suggested that the beneficial effects of EOs containing mainly CVL were exerted through the modulation of gut microbiota. [71]. Ceppa et al. [119] observed that EOs mixture of oregano and thyme had a modulatory effect on gut microbiota in rainbow trout (*Oncorhynchus mykiss*). In a mouse model of antibiotic-associated gut dysbiosis and *Clostridium difficile* infection, CVL supplementation significantly reduced the incidence of diarrhea and improved gut dysbiosis. CVL had a positive effect on microbiome composition by enhancing the abundance of beneficial bacteria such, as *Firmicutes*, and reducing the proportion of harmful bacteria such as *Proteobacteria* [75]. These effects could also be attributed to the direct antibacterial properties of CVL against pathogenic bacteria. Infection with *Clostridium perfringens* can cause necrotic enteritis. In a *C. perfringens* infected broiler chicken model, EO supplementation (25% thymol, 25% carvacrol) alleviated gut lesions and enhanced serum antibody titers against the Newcastle disease virus. In the ileum, EOs increased the expression of occludin mRNA and inhibited TLR2 and TNF-α expression [76]. Additionally, supplementation with EOs resulted in changes in the host ileal microbial population—an increase in *Lactobacillus crispatus* and *Lactobacillus agilis* accompanied by a decrease in *Lactobacillus salivarius* and *Lactobacillus johnsonii* [77]. In a duck model, supplementation with EOs primarily containing thymol and carvacrol decreased microbial populations of coliforms, total aerobes, and lactose-negative *Enterobacteria* [72]. In addition to the direct immunostimulatory effects of EOs containing thymol and carvacrol demonstrated in a Juvenile hybrid tilapia animal model [78], EOs exerted an indirect effect through changes mediated by the microbiota. This is backed up by the observation that the treatment of germ-free zebrafish colonized by microbiota with EOs exerted a suppressive effect on levels of inflammatory markers such as serum amyloid and interleukin 1β, 8 and upregulated the expression of claudin-1 and occludin-2, two important tight junction proteins for intestinal permeability and integrity [78]. In a model using human intestinal epithelial cells Caco-2, CVL treatment resulted in a significant reduction of *C. jejuni* adhesion, invasion, and translocation to Caco-2 cells, as well as a reduction in *C. jejuni* motility, toxin production, and pathogenic gene expression [79]. Mooyotto et al. showed that CVL reduced *C. difficile* toxin production using an in vitro bacterial culture method and reduced *C. difficile* cytotoxicity using Vero cells [120] CVL was also able to reduce the invasive ability of *Salmonella typhimurium* in intestinal epithelial cells IPEC-J2 and inhibit bacterial viability and motility in vitro [121]. Additionally, CVL increased the relative abundance of *Lactobacillus* spp. in the chicken gut [80]. In a murine infection model of acute *campylobacteriosis*, CVL reduced disease symptoms by reducing intestinal apoptosis and pro-inflammatory immune responses not only in the intestine, but also in extra-intestinal organs such as the liver, as indicated by decreased serum levels of IFN-γ, TNF, MCP-1 and IL-6 [81]. Michiels et al., showed that CVL and thymol improved gut health in weaning piglets by reducing the number of intra-epithelial lymphocytes and concurrently increasing the villus height to crypt depth ratio in the distal small intestine [122]. Moreover, supplementation with carvacrol and thymol in weaning pigs reduced the intestinal oxidative stress caused by weaning, increased the *Lactobacillus* population, and decreased the number of *Enterococcus* and *E. coli* in the jejunum along with a significant decrease in the expression of TNF-α [83]. Another study showed that the supplementation of EOs containing CVL and thymol in pigs resulted in increased nitrogen digestibility and decreased emission of ammonia and total fecal nitrogen. Additionally, microbial protease and urease activities were inhibited by EO supplementation [123]. Recently, an integrative study investigating the transcriptional response and microbiota modulation in the intestine in response to a diet enriched with carvacrol and thymol essential oils was conducted using a gilthead seabream (*sparus aurata*) animal model [124]. Several remarkable changes in inflammation and the expression of immunity genes were observed. The activation of the NF-kB pathway, which could impair the maintenance of intestinal epithelial integrity and immune homeostasis, was inhibited. The fish supplemented with EOs also had downregulated expression of NF-kB mediated proinflammatory cytokines such as interleukin-1 beta (il-1β) [124]. Using chicken and mice models, CVL supplementation prevented *Campylobacter* (a common foodborne pathogen) infection and colonization by reducing the expression of a vast number of genes involved in motility, adhesion, growth, metabolism, and anaerobic respiration [82]. In a model using human intestinal epithelial cells Caco-2, CVL treatment resulted in a significant reduction of *C. jejuni* adhesion, invasion, and translocation to Caco-2 cells, as well as a reduction in *C. jejuni* motility, toxin production and pathogenic gene expression [79]. In another cellular study, Mooyotto et al., showed that CVL reduced *C. difficile* toxin production using an in vitro bacterial culture method and reduced *C. difficile* cytotoxicity using Vero cells [120] CVL was also able to reduce the invasive ability of *Salmonella typhimurium* in intestinal epithelial cells IPEC-J2 and inhibit bacterial viability and motility in vitro [121].

#### 3.2.3. Rosmarinic Acid

Rosmarinic acid (RA) is a phenolic acid commonly found in the *Lamiaceae* plant species. RA is the most abundant phenolic acid present in *O. syriacum* and *T. spicata*. Alongside antioxidant and anti-inflammatory effects in several diseases, RA demonstrated a protective effect within in vitro and in vivo models of gastric mucosal injury [73]. RA exhibited cytoprotective, antioxidant, anti-apoptotic, and wound healing properties in the gastric mucosal epithelial cell line RGM-1 insulted with 5% ethanol. The results were further validated in vivo with ethanol-induced gastric mucosal lesions in mice, where pre-treatment with extract rich in RA promoted gastric mucosal healing by decreasing oxidative stress, inflammatory response, proapoptotic protein expression, and gastric mucosal damage. In addition, the group treated with extract rich in RA showed higher microbial diversity when compared to the control group. In particular, an increase in *Muribaculaceae* and *Ruminococcaceae* and a decrease in *Prevotellaceae* was observed in mice treated with RA-rich extract. Additionally, levels of SCFAs such as acetic acid and propionic acid were slightly increased, and butyric acid was notably increased in RA-treated mice compared to control mice [73]. In another study, the prebiotic effects of RA on gut microbiota were demonstrated by an increase in the population of diabetes-resistant bacteria and a decrease in diabetes-sensitive bacteria in diabetic rats [74]. 

#### 3.2.4. Oleanolic and Ursolic Acid

Oleanolic acid (OA) and ursolic acid (UA) are pentacyclic terpenoids with pharmacologic properties found in olive oil and medicinal herbs such as *O. syracum* [125]. OA and UA show antibacterial and antiparasitic activity in the gastrointestinal tract [126]. OA and UA inhibited *E. coli* enterotoxin-induced diarrhea in mice by blocking the binding of enterotoxins to the surface of intestinal epithelial cells [127]. Oral administration of OA in a mice model of colitis significantly inhibited colon shortening and myeloperoxidase activity, displayed potent anti-inflammatory properties by reducing the activation of TNF-α, IL-1β, IL-17, NF-κB, and MAPK, and increasing the expression of IL-10. The study also showed that OA protected intestinal integrity by increasing the expression of tight junction proteins ZO-1, occludin, and claudin-1 in the colon [84]. Similarly, UA exerted anti-inflammatory and antioxidant effects in mice with ulcerative colitis [85,86], and regulated intestinal microbiota composition and inflammatory cell infiltration [87]. UA also displayed protective effects on the intestinal mucosal barrier by attenuating intestinal injury an ileal epithelial cells apoptosis, decreasing LPS, procalcitonin, and intestinal malondialdehyde levels, and increasing the expression of the tight junction proteins claudin 1 and occludin in the ileum of rats [128]. In a mice model of liver fibrosis, UA reduced intestinal damage by inhibiting TNF-α, increasing the expression of tight junction proteins ZO-1 and occludin, as well as intestinal antimicrobial peptide angiogenin-1 to protect the gut barrier. Considerable effects of UA were observed in an antibiotic-resistant mice model. Treatment with UA protected the intestinal barrier by increasing the height of jejunal villi, decreasing jejunal crypt depth, and upregulating the expression of tight junction proteins ZO-1, claudin-1 and occludin. Additionally, UA decreased serum LPS and diamine oxidase levels and downregulated the expression of pro-inflammatory TNF-α and IL-6 cytokines. Supplementation with UA had beneficial effects on gut microbiota composition, resulting in a decrease in *Firmicutes* and *Ruminococcaceae*, an increase in *Bacteroidetes*, and enhanced the growth of SCFA-producing bacteria including the *Rikenellaceae* and *Bifidobacteriaceae* families [88]. Additionally, UA treatment improved gut dysbiosis by increasing *Lactobacillus* and *Bifidobacterium* populations [89,90].

UA modified the gut microbiota by promoting the growth of *Lactobacillus*, inhibiting the proliferation of harmful bacteria such as *Burkholderiales, Alphaproteobacteria*, *Betaproteobacteria*, and *Gammaproteobacteria*, as well as downregulating the expression of antibiotic resistance genes [91]. 

OA displayed a direct effect on intestinal tight junctions and inflammation in a mice model with *Salmonella typhimurium*-induced diarrhea. OA alleviated intestinal damage and maintained gut barrier integrity by enhancing the expression and localization of occludin, claudin-1, and ZO-1. OA displayed its anti-inflammatory potential by reducing the levels of standard inflammatory markers such as COX-2, iNOS, pro-inflammatory cytokines IL-1β, IL-6, and TNF-α, as well as the physphorylation and degradation of IκB, nuclear translocation of p65, TLR4, and the activation of the MAPK pathway [92]. OA prevented gut atrophy induced by parenteral nutrition in pigs. OA is an agonist of the bile acid-activated G protein-coupled receptor TGR5. The activation of TGR5 by OA ameliorated gut atrophy [93]. Recently, the role of OA in the gut-liver axis was investigated by Xue et al. in HFD rats with interesting results. In addition to combatting obesity and hepatic steatosis, OA supplementation attenuated HFD-induced metabolic endotoxemia and intestinal barrier damage by decreasing LPS and pro-inflammatory cytokines. OA also displayed an intestinal protective effect by increasing the expression of intestinal tight junction proteins ZO-1 and occludin, decreasing serum diamine oxidase (DAO) activity and d-lactate concentrations, and reducing intestinal inflammation by inhibiting the TLR4/NF-κB pathway [94]. The authors found that OA could modulate gut microbiota and improve gut immunity by enhancing microbial diversity, reducing the ratio of *Firmicutes* to *Bacteroidetes*, and increasing the abundance of butyrate-producing bacteria [94,95]. In addition, oleanolic acid glycosides (3-O-glycoside moiety) accelerated gastrointestinal transit and prevented ileus in mice [129]. UA-modulated sphingomyelinase (SMase) activity, is associated with several inflammatory diseases and tumors including inflammatory bowel disease and colon cancer [130,131].

#### 3.2.5. *Rhus coriaria* (Sumac)

Plants belonging to the *Rhus* genus have been commonly used in traditional medicine to treat gastrointestinal disorders [27,51]. Several *Rhus* species that share various phytochemicals with *R. coriaria* have demonstrated beneficial effects in intestinal disorders in animal and cellular models of gastrointestinal inflammation [132,133], diarrhea [134,135], ulcerative colitis [136], and *Vibrio vulnificus* infection [137]. In streptozotocin-induced diabetic rats with intestinal oxidative damage, supplementation with sumac extract enhanced the antioxidant defense system by increasing the level of GSH, GST, GR, CAT, GPx, and SOD in the small intestine, and decreasing MDA levels and α-glucosidase activity [97]. In a necrotizing enterocolitis model using newborn rat pups, Isik et al. evaluated the protective effects of sumac supplementation on intestinal injury. Sumac ameliorated histopathologic and biochemical markers in the ileum and proximal colon, and also showed anti-apoptotic and anti-inflammatory effects in the rat colon. Additionally, sumac reduced oxidative stress by reducing lipid peroxidation and DNA and protein oxidation [98]. The inhibitory effect of sumac on the enzyme urease was tested in vitro. Urease is essential for the colonization of the gastric mucosa by *Helicobacter pylori*, a bacterium that induces gastrointestinal diseases such as gastritis and peptic ulcer disease, potentially leading to gastric cancer. Sumac showed a potent inhibitory effect against Jack bean urease activity [138]. The effects of sumac on microbiota were evaluated by using a metabolomic approach in an in vitro model of gut microbiota. Farag et al. investigated the response of the gut microbiota to sumac by analyzing changes in metabolites in bacterial cultures. Overall, a decrease in amino acid levels, nitrogenous compounds, and sugar levels was observed in samples treated with sumac. These changes were accompanied by an increase in levels of SCFA and nucleic acids [96].

Gallic acid (GA), the most abundant phenolic compound in sumac, and plant extract rich in gallic acid showed potential prebiotic effects associated with a decrease in intestinal inflammation and the promotion of intestinal integrity [139]. A recent study reported that GA may have a protective effect against colon toxicity. Colon toxicity induced by 1,2-dimethylhydrazine in female Waster rats was characterized by increased inflammation, apoptosis, oxidative stress, goblet cell disintegration, and mucin depletion. Treatment with GA reduced all these parameters and increased glutathione content and the activity of the detoxifying enzymes GPx, GR, GST, and CAT [101]. Several studies have reported that GA exerted inhibitory effects on intestinal pathogenic bacteria, such as *Clostridium histolyticum*, *Clostridium difficile*, and *Bacteroides* spp., which have been associated with several intestinal diseases [140]. Penta-O-galloyl-β-D-glucose—a gallotannin present in the *Rhus* plant family and sumac-inhibited NF-κB and MAPK activation in stimulated peritoneal and colonic macrophages and suppressed IL-1β, TNF-α, and IL-6 in LPS-stimulated peritoneal macrophages, while increasing the expression of the anti-inflammatory cytokine IL-10 in vitro. In a mice model of colitis, supplementation with penta-O-galloyl-β-D-glucose inhibited colon shortening and myeloperoxidase activity, reduced the activation of NF-κB and levels of IL-1β, TNF-α, and IL-6, but increased IL-10 levels [99]. 

In addition, GA showed a protective effect against ulcerative colitis mediated by gut microbiota g modulation in the rat. Treatment with GA increased the growth of probiotic bacteria, such as *Lactobacillaceae* and *Prevotellaceae*, and decreased the number of several pathogenic species, such as *Firmicutes* and *Proteobacteria* families. GA also induced metabolic changes by increasing carbohydrate bile acid metabolism and decreasing amino acid metabolism [100]. A mixture of GA and anthocyanins, which are compounds abundant in sumac, significantly enhanced the growth of *Bifidobacterium* spp. and *Lactobacillus* spp. [141]. Anthocyanins are primarily transformed into GA by the gut microbiota, contributing to their anti-inflammatory effects [142].

To summarize, the potential bioactivity of GA, GA-rich extracts, and GA metabolites from gallotannins and anthocyanins against inflammatory disorders and intestinal disease, as well as their potential for microbiota modulation, have been recently extensively reviewed [139,143,144]. The evidence available so far suggests that along with its direct antioxidant and anti-inflammatory effects, GA may modulate the gastrointestinal immune system by modifying the composition of gut microbiota. 

#### 3.2.6. *Sesamum indicum* (Sesame)

Sesame sauce displayed anti-inflammatory and anti-cancerogenic effects in a mouse colon carcinogenesis model. Specifically, supplementation with sesame sauce decreased the expression and serum levels of tumor necrosis factor-α, interferon-γ, interleukin (IL)-6, IL-17α, inducible nitric oxide synthase, and cyclooxygenase-2 in mice colon mucosa [102]. Sesamol, a lignan present in sesame oil with antioxidant and anti-inflammatory properties, suppressed cyclooxygenase-2 transcriptional activity in colon cancer cells and modified the development of intestinal polyps in mice [145]. Sesamol protected gut barrier integrity and reduced the release of LPS in aging mice and HFD models. Treatment with sesamol increased the length of intestinal villi and muscularis mucosa thickness and elevated mRNA expression of tight junction complex claudin-1 in the colon. Moreover, sesamol was able to attenuate HDF-induced inflammation in the colon by reducing the expression of IL-1β and TNF-α [103,104]. Sesaminol glucosides alleviated premalignant lesions of the rat colon [146]. These findings indicate that sesame may possess chemopreventive properties.

Cecal ligation and puncture is a well-established model for abdominal sepsis. In septic rats and mice sesame oil significantly decreased markers of oxidative stress such as lipid peroxidation and serum nitrite levels, consequently attenuating hepatic injury. Moreover, supplementation with sesame oil increased the survival rate and levels of anti-inflammatory cytokine IL-10, as well as significantly reduced xanthine oxidase activity [147,148,149,150]. Rezaeipour et al. observed that diets containing sesame meal increased villus height and villus height to crypt depth ratio in the jejunum [151]. In a rat model of chemically-induced acute colitis, sesame oil displayed potential healing effects as shown by a significant decrease in the levels of inflammatory markers (mast cells and CD68+ cells), fibrosis (collagen, laminin), and modulation of colon mucins by a decrease in acidic mucin and increase in neutral mucin [106]. Similar results were also observed in colitic mice, with a fermented sauce containing sesame displaying significant protective and anti-inflammatory effects in the colon [152]. In a rat model of acetic acid-induced inflammatory bowel disease, the level of myeloperoxidase, lipid peroxidation, and nitrite in the colon was reduced following sesamol treatment [107]. Sesamol showed a protective effect against radiation-induced gastrointestinal injury in mice, with pre-treatment with a single dose of sesamol partially decreasing radiation-induced mortality. Additionally, pre-treatment with sesamol decreased lipid peroxidation and the translocation of gut bacteria to the spleen and liver and enhanced the regeneration of crypt cells in the gastrointestinal tract [108]. The effect of sesame constituents on gut microbiota is well observed. A significant increase in microbiome diversity was observed in the group supplemented with sesamol, with higher numbers of *Bifidobacterium* and *Akkermansia* and lower numbers of *Clostridium* bacteria, when compared to aging mice [104]. In HFD mice, sesamol significantly improved the relative abundance of *Bacillales*, *Fusobacterium*, and *Lactococus*, while decreasing the number of *Bilophila*. In addition, sesamol significantly increased the level of SCFAs such as acetate, propionate, and butyrate [103]. Likewise, supplementation with sesamol in an Alzheimer mice model altered gut microbiota by significantly decreasing the relative abundance of *Helicobacter hepaticus* and *Clostridium* spp. and increasing the relative abundance of *Rikenellaceae* and *Bifidobacterium* [105]. The authors showed that sesamol also has a protective effect on gut barrier integrity, decreasing LPS leakage into the serum and increasing levels of SCFAs, including acetate, propionate, isobutyrate, butyrate, and valerate [105]. These findings indicate that sesamol can decrease systemic inflammation and improve brain disease by protecting the integrity of the gut barrier, altering the gut microbiota, reducing LPS levels, and increasing the level of SCFAs in the blood.

### 3.3. Clinical Studies (Table 3)

The effect of a diet rich in polyphenols on increased intestinal permeability was investigated by measuring serum zonulin levels in a randomized, controlled, cross-over clinical trial [153]. Polyphenol intake significantly decreased serum zonulin levels and blood pressure. This effect was accompanied by a significant increase in fiber-fermenting and butyrate-producing bacteria such as *Ruminococcaceae* and *Faecalibacterium*. Notably, the effect of a polyphenol-rich diet was greater in subjects with metabolic syndrome, suggesting an association between intestinal permeability and metabolic syndrome [153]. The polyphenol-rich diet used in the study shares a similar profile with the polyphenolic compounds present in the Za’atar mixture. Za’atar contains high concentrations of polyphenols and ursolic and oleanolic acids, which have also been associated with the improvement of several components of metabolic syndrome in humans such as the reduction of inflammatory cytokines, plasma lipid and cholesterol levels [154,155]. Gallotannins and gallic acid-rich extract (which are also found in high amounts in sumac and Za’atar mixture) improved chronic constipation, increased levels of gastrin and valeric acid, and decreased levels of endotoxins and interleukin 6 [156]. After consuming Gallotannins and gallic acid-rich mango extract for six weeks, lean and obese individuals had increased levels of tannase-producing *Lactococcus lactis* and decreased levels of *Clostridium leptum* and *Bacteroides thetaiotaomicron*, which have been shown to be associated with obesity. In addition, an increase in fecal SCFAs, such as butyric and valeric acid, and a decrease in endotoxin levels were observed after the consumption of polyphenols [157]. Polyphenols have also demonstrated anti-inflammatory and gut microbiota-modifying effects in IBD patients. Gallotannins and gallic acid-rich mango pulp intake (200–400 g) for eight weeks reduced plasma levels of pro-inflammatory cytokines such as interleukin 8 (IL-8), growth-regulated oncogene and granulocyte-macrophage colony-stimulating factor. The consumption of polyphenols modulates the composition of gut microbiota by increasing the abundance of *Lactobacillus plantarum*, *Lactobacillus reuteri,* and *Lactobacillus lactis*, accompanied by increased fecal butyric acid production [139].

A randomized, placebo-controlled, crossover study showed that sesame consumption improved blood lipids and exerted antioxidant effects [158]. In addition, in subjects with partial adhesive small bowel obstruction, treatment with sesame oil had a positive effect on clinical parameters including a decreased need for surgical intervention, and a shorter convalescence and hospital stay compared to the control group [159].

**Table 3:** Clinical studies assessing the effects of a polyphenol-rich diet on gut microbiota and intestinal permeability in gastrointestinal disorders.

Authors (Year)	Sample Size	Gender M/F (Age)	Participants	Format, Dose	Duration of Study	Main Findings
[153]	51	27/39 (≥60 years)	Subjects with increased IP	PR-diet 724 mg/day	8 weeks	↓ Serum zonulin ↓ Blood pressure ↑ Fiber-fermenting bacteria, butyrate-producing bacteria
[156]	36	11/25 (8–65 years)	Subjects with chronic constipation	Mango consumption, 300 g/day	4 weeks	↑ Stool frequency, consistency ↑ Gastrin levels, SCFA (valeric acid) ↓ Endotoxin and IL-6
[157]	32	22/10 (18–50 years)	healthy lean and obese individuals	Mango consumption, 400 g/day	6 weeks	↑ SCFA in lean individuals
[139]	10	5/5 (18–75 years)	IBD Subjects	Mango pulp intake, 200–400 g/day	8 weeks	↑ SCCAI ↓ IL-8 ↓ GRO and GM-CSF ↑ Fecal butyric acid ↓ Inflammation biomarkers ↑ *Lactobacillus plantarum, Lactobacillus reuteri, Lactobacillus lactis*
[159]	64	33/31 (19–79)	Subjects with symptoms and signs of SBO	Sesame oil in nasogastric tube, 150 mL/day	1 day	↓ SBO resolution time ↓ Hospital stays ↓ Relaparotomy rate

**Abbreviations:** PR-diet—polyphenol-rich diet; IP—intestinal permeability; SCFAs—short-chain fatty acids; IL—interleukin; GRO—growth-regulated oncogene, GM-CSF—colony-stimulating factor; SBO—small bowel obstruction; SSCAI—squamous cell carcinoma antigen-1; IBD—Inflammatory bowel diseases; ↑—increased; ↓—decreased.

## 4. Summary

A growing number of studies have reported potential gut microbiota-mediated effects of plant-based functional foods rich in polyphenols on human health and disease. Moreover, plant-derived polyphenols have attracted much attention in regard to the regulation of intestinal barrier function [160]. In humans, the bioactivation of polyphenols happens through intestinal transformation and is mediated partly by digestive enzymes, but fundamentally by gut microbiota. Once the active “smaller” phenolic compounds and metabolites are absorbed into the portal vein, they travel to various tissues and organs to enact their beneficial role [161]. However, polyphenols also exert local cytoprotective and antioxidant effects, especially on intestinal cells. The plant-based Za’atar mixture is widely used as a food and ingredient in Lebanon and the Eastern part of the Mediterranean basin. The mixture is rich in dietary fiber and phenolic compounds, as well as minerals and vitamins. Za’atar mixture is mainly a blend of *O. syriacum* (known as the Za’atar plant in Lebanon) and *Thymbra spicata* (wild Za’atar), *R. coriaria* (sumac), and sesame. Despite its richness in bioactive compounds and its nutraceutical potential, studies on the whole Za’atar mixture are still missing, however, most of the studies have focused on the single components or single (poly)phenolic compounds. Here we reviewed the literature available on chemical and phytochemical profile of Za’atar, and its combined effects on the gastrointestinal tract.

Studies have shown that even at low doses the components present in Za’atar are able to prevent and reduce chemically induced acute inflammation in animal models. In addition, these compounds have exhibited protective and therapeutic effects in pathogen and chemical-induced colitis. Furthermore, polyphenols can attenuate the mucosal activation of NF-κB, the production of cytokines, intestinal barrier dysfunction, and apoptosis (Table 2). 

Gut dysbiosis is closely related to increased intestinal permeability. Therefore, the modulation of gut microbiota can have a positive effect on intestinal permeability and *vice versa*. Diet can play a modulatory role, although the exact molecular mechanisms involved in this interplay remain unclear, especially in humans [162]. 

**Figure 3:**
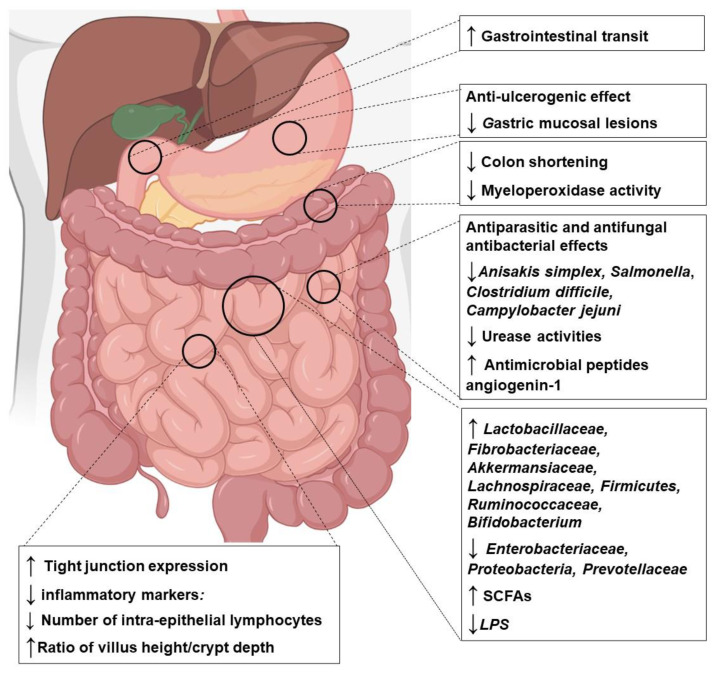
Summary of the combined effects of polyphenols found in Za’atar components on the gastrointestinal tract. Abbreviations: SCFA—short-chain fatty acids; LPS—lipopolysaccharide; ↑—increased; ↓—decreased. The figure is developed using https://biorender.com/ (accessed on 12 December 2022).

As shown in Table 2 and Table 3, several experimental studies in animals and humans have reported that Za’atar components may exert prebiotic-like effects on gut microbiota by enhancing the growth of beneficial bacteria (e.g., *Lactobacillus* spp., *Bifidobacterium* spp., fiber-fermenting, and butyrate-producing bacteria) and reducing the relative number of potentially harmful and pathogenic bacteria (e.g., *Clostridium* spp.). Additionally, Za’atar components may be beneficial in the treatment of intestinal inflammatory diseases and leaky gut syndrome. Numerous studies have reported the potential antioxidant and anti-inflammatory effects of Za’atar compounds in experimental models of intestinal disease in animals and humans (Table 2 and Table 3). Based on a review of the available studies, we show that Za’atar compounds may significantly reduce intestinal permeability by increasing the level of tight junction proteins and by maintaining gut epithelial integrity. The overall possible effects of Za’atar on gastrointestinal tract are depicted in the Figure 3.

## 5. Clinical Perspectives and Future Applications

The use of natural compounds in the prevention and treatment of disease has garnered increasing clinical and medical interest over the past decades.

The idea of natural compounds is appealing due to their ease of use as dietary supplements, lower associated costs, and a more favorable side effect profile when compared to synthetic drugs. Another attractive aspect is the potential use of natural compounds in the treatment of functional gastrointestinal disorders (e.g., dyspepsia, irritable bowel syndrome, constipation) as an adjunct to dietary intervention and lifestyle modifications. In fact, individuals affected by functional gastrointestinal disorders often seek out alternative methods to improve their symptoms when the limited number of currently available guideline-based treatment options have failed or provided insufficient improvement of symptoms [163]. Factors implicated in functional gastrointestinal disorders include gastrointestinal dysmotility, altered intestinal barrier and immune function, dysbiosis, dysregulation of neurologic and signaling networks, and stress [164]. The effects of Za’atar components reported so far are related to several of these factors, raising questions as to whether Za’atar and its components might have beneficial clinical effects.

Although the potential clinical applications of natural compounds such as Za’atar are attractive, research on this mixture and its components is still in its early stages before evidence-based conclusions and recommendations can be made. We cover the advantages and limitations of Za’atar use in the following paragraphs.

Advantages: Za’atar is a low-cost and environmentally friendly plant-based food consisting of locally available herbs. Active compounds found in Za’atar have gained attention due to their potential role in the prevention and treatment of cardiovascular, metabolic, and gastrointestinal diseases with no clinically significant side effects reported in studies undertaken so far. Based on our previous discussion on the properties of its compounds, we raise the following questions on whether Za’atar could be applied as: (i) a prebiotic-like agent, (ii) an adjunct to dietary modifications used in the prevention or treatment of metabolic and gastrointestinal diseases; (iii) a supplement and nutraceutical.

Limitations: Despite the health-promoting effects of the components of Za’atar described previously, an understanding of the biological effects of the Za’atar mixture as a whole is missing. More animal and human studies are necessary to further elucidate the translational aspects of the effects of Za’atar and to fully characterize the mixture as a preventive and therapeutic tool to ascertain its viability as a plant-based functional food. In fact, there is no defined therapeutic dose for the Za’atar mixture, which could partially explain why there is a lack of clinical trials. 

Suggestions concerning and covering the general quality and safety aspects of traditional herbal medicine or food products are depicted in the following points:

1. Well-designed human studies evaluating drug interaction with herbal supplements are required.
2. Controlled clinical trials evaluating the acceptance, tolerance, and safety of herbal preparation are highly needed.
3. Standardized preparations of herbal products or traditional foods should be adjusted to an exact content of substances with well-known nutraceutical properties.

Regarding the possible effects on the gastrointestinal tract, future clinical trials investigating Za’atar mixture should consider the following: (i) consider the interaction of Za’atar with gut microbiota and levels of short-chain fatty acids (SCFAs); (ii) take into account the effect of Za’atar on intestinal barrier function and selectivity by assessing levels of oxidative stress, inflammatory markers, and tight junction proteins; (iii) interpret the effects observed in the context of dietary factors as well as an adjunct to the appropriate dietary intervention.

In this context, our future molecular and clinical studies on gastrointestinal motility, intestinal permeability, and microbiota composition and metabolites will address the effects of the Za’atar mixture whilst testing its clinical efficacy in a selected group of subjects, with the aim of evaluating the potential health-promoting effects of Za’atar.

## 6. Conclusions

Several studies on cellular and animal models, as well as in humans, have demonstrated the beneficial effects of individual plant-based bioactive compounds on the gastrointestinal tract. The combination of well-known medicinal and health-promoting herbs in Za’atar could provide synergistic pharmacological and nutraceutical effects, especially pertaining to the gastrointestinal tract and gut microbiota. In conclusion, a Za’atar mixture rich in polyphenols, essential oils, and fiber could provide beneficial effects in the modulation of gut microbiota and intestinal permeability. Its richness in bioactive compounds, safety, and prebiotic properties make mixed Za’atar a potential promising adjunct to existing treatments used in gastrointestinal and metabolic diseases. However, studies of the effects of the whole Za’atar mixture are required to explore the combined effects and safety of Za’atar, in addition, well-controlled human studies are necessary to improve our understanding of the molecular mechanisms and effects on microbiota and intestinal permeability before any conclusions can be made on the clinical application of Za’atar.

## Figures and Tables

**Table 2:** Summary of in vitro and in vivo studies on the effects of Za’atar bioactive compounds on gastrointestinal disorders and gut microbiota.

Plants	Sample Used	Model	Disorder	Main Funding	Refs.
*O. syracium* and derived compounds	EOs	In vitro *Anisakis simplex* Parasite	Gastrointestinal Parasite	↑ Parasite mortality ↓ Penetration ability	[68]
	Extract	In vivo Ethanol-induced ulcer in Swiss albino mice	Peptic ulcer	↓ Ulcer score ↓ Gastric damage	[69]
	EOs	In vivo Pigs	Microbiota	↑ *Bacilli, Lactobacillales, Streptococcaceae, Veillonellaceae*	[70]
	OEO	In vivo Pigs	Microbiota	↑ *Lactobacillaceae, Fibrobacteriaceae,* *Akkermansiaceae,* *Lachnospiraceae* ↓ *Enterobacteriaceae*	[71]
	OEO	In vivo Ducks	Microbiota-performance	↓ Coliforms, total aerobes, lactose-negative *Enterobacteria*	[72]
	RA	In vivo Ethanol-induced gastric mucosal injury in Male BALB/c mice	Peptic ulcer	↓ Oxidative stress ↓ Inflammation ↓ Apoptosis	[73]
	RA-rich extract	In vivo Male BALB/c mice	Microbiota	↑ *Muribaculaceae* and *Ruminococcaceae* families ↓ *Prevotellaceae* family ↑ SCFAs	[73]
	RA	In vivo Streptozotocin-induced diabetic rats	Diabetes-Microbiota	↑ *Actinobacteria,* *Bacteroides, Faecalibacterium, Lachnospiraceae, Prevotella*	[74]
	CVL	In vivo *Clostridium difficile*-infected C57BL/6 mice	Gut dysbiosis-Microbiota	↓ Diarrhea ↑ *Firmicutes* ↓ *Proteobacteria*	[75]
	CVL-Thymol	In vivo *C. perfringens*-infected broiler chickens	Necrotic enteritis	↓ Gut lesions ↓ TLR2, TNF-α ↑ Occludin ↑ *Lactobacillus crispatus, Lactobacillus agilis* ↓ *Lactobacillus salivarius*, *Lactobacillus johnsonii*	[76,77]
	EO	In vivo Hybrid Tilapia-Germ Free Zebrafish	Immunity-Microbiota	↓ IL-1β, IL-8 ↑ Claudin1, Occludin2	[78]
	CVL	In vivo Chickens and mice In vitro Caco2 cells	*Campylobacter jejuni* infection (campylobacteriosis)	↓ *C. jejuni* adhesion, invasion, and translocation ↑ *Lactobacillus* spp ↓ Inflammation ↓ Apoptosis ↓ IFN-γ, TNF, MCP-1 and IL-6	[79,80,81,82]
	CVL-thymol blend	In vivo Weaning piglets	Gut disorders	↓ Oxidative stress markers ↑ *Lactobacillus* genus ↓ *Enterococcus* genus	[83]
	Oleanolic acid Ursolic acid	In vivo Mice	Colitis	↓ NF-κB and MAPK activation pathway ↓ IL-1β, TNF-α, IL-6 ↑ IL-10 ↓ Colon shortening, myeloperoxidase activity ↑ ZO-1, occludin, claudin-1	[84,85,86,87]
	Ursolic acid	In vivo Hamster	Hypercholesterolemia-Gut Microbiota	↓ Intestinal cholesterol absorption ↓ *Firmicutes, Ruminococcaceae* ↑ *Bacteroidetes*, *Rikenellaceae*, *Bifidobacteriaceae*	[88]
	Ursolic acid	In vivo Mice	Liver fibrosis-intestinal damage	↓ TNF-α, MDA, LPS ↑ ZO-1, occludin ↑ Intestinal antimicrobial peptides, angiogenin-1 ↑ *Lactobacillus, Bifidobacterium, Ruminiclostridium*	[89,90]
	Ursolic acid	In vivo Mice	Antibiotic Resistance-Microbiota	↓ TNF-α, IL-6, LPS, DAO ↑ ZO-1, occludin ↑ *Lactobacillus,* ↓ *Burkholderiales, Alphaproteobacteria, Betaproteobacteria, Gammaproteobacteria*	[91]
	Oleanolic acid	In vivo Mice	Diarrhea-intestinal inflammation	↑ ZO-1, occluding ↓ Intestinal damage ↓ NF-κB and MAPK activation pathway ↓ IL-1β, TNF-α, IL-6	[92]
	Oleanolic acid	In vivo Pigs	Gut atrophy	↑ Gut mass ↑ Villous/crypt ratio ↑ TGR5 expression	[93]
	Oleanolic acid	In vivo Rats	MetS-intestine damage-Microbiota	↓ LPS, DAO, d-lactate ↑ ZO-1, occluding ↓ Intestinal damage ↓ NF-κB ↓ IL-1β, TNF-α, IL-6 ↓ *Firmicutes/* *Bacteroidetes* ratio ↑ *Ruminiclostridium, Ruminococcaceae*	[94,95]
*Rhus coriaria* and derived compounds	Extract	In vitro Bacterial microbita culture	Microbiota	↑ SCFA ↑ Fructose	[96]
	Extract	In vivo Streptozotocin (STZ)-induced diabetic rats	Diabetes-gut damage	↓ Blood glucose ↓ TG, TC ↓ AST, ALT, LDH, ALP, MDA	[97]
	extract	In vitro Rat pups	necrotizing enterocolitis	↓ Oxidative stress markers ↓ Apoptosis ↓ Inflammation	[98]
	Penta-O-galloyl-β-D-glucose	In vivo Rats	Colitis	↓ NF-κB and MAPK activation pathway ↓ IL-1β, TNF-α, IL-6 ↑ IL-10 ↓ Colon shortening, myeloperoxidase activity	[99]
	GA	In vivo Rats	ulcerative colitis	↑ *Lactobacillaceae, Prevotellaceae* ↓ *Firmicutes, Proteobacteria* ↑ Carbohydrate and bile acid metabolism ↓ Amino acid metabolism	[100]
	GA	In vivo Rats	Colon toxicity	↓ Oxidative stress markers ↓ Apoptosis ↓ Inflammation ↓ Goblet cell disintegration ↓ Mucin depletion ↑ GPx, GR, GST, CAT, GSH	[101]
Sesame and derived compounds	Sesame sauce	In vivo Mice	Colonic Carcinogenesis	↓ TNF-α, IL-6, IF-γ, IL-17α, iNOS, COX-2	[102]
	Sesamol	In vivo Mice	Intestinal integrity-Microbiota	↑ Gut barrier integrity ↓ LPS release ↓ TNF-α, IL-6 ↑ *Bacillales, Fusobacterium Lactococus* ↑ SCFAs	[103]
	Sesamol	In vivo Mice	Aging-systemic inflammation	↑ Gut barrier integrity ↓ LPS release ↑ *Bifidobacterium, Akkermansia* ↓ *Clostridium*	[104]
	Sesamol	In vivo Mice	Alzheimer-Intestinal Integrity-Microbiota	↑ Gut barrier integrity ↓ LPS release ↑ Gut barrier integrity ↓ LPS release ↓ *Helicobacter hepaticus, Clostridium* ↑ *Rikenellaceae, Bifidobacterium*	[105]
	Sesame oil	In vivo Rats	Acute colitis	↓ Colitis index ↓ Inflammation ↓ Fibrosis ↓ Acidic mucin ↑ Natural mucin	[106]
	Sesamol	In vivo Rats	Inflammatory bowel disorders	↓ MPO, MDA. NO	[107]
	Sesamol	In vivo Mice	Gastrointestinal injury	↓ Mortality ↓ Lipid peroxidation ↓ Apoptosis ↑ Regeneration of crypt cells ↓ Gut bacteria translocation	[108]

**Abbreviation:** Eos—essential oils; SCFAs—short-chain fatty acids; OEO—oregano essential oil; RA—rosmarinic acid; MPO—myeloperoxidase; NO—nitric oxide; DAO—diamine oxidase; MetS-metabolic syndrome; CVL—carvacrol; GA—gallic acid; TLR-2—toll-like receptor 2; TNF—tumor necrosis factor; IL—Interleukin; MCP-1—Monocyte Chemoattractant Protein-1; NFkB—nuclear factor kappa b; MAPK—Mitogen-activated protein kinases; ZO-1—Peripheral membrane protein; STZ—streptozotocin; LPS—lipopolysaccharide; DAO—diamine oxidase; TGR5—G protein-coupled bile acid receptor; TG—triglyceride; TC—total cholesterol; AST—glutamic oxalacetic transaminase; ALP—alkaline phosphatase; LDH—lactate dehydrogenase; CAT—catalase; GR—glutathione reductase, GPx—glutathione peroxidase, GST—glutathione-S-transferase; GSH: Glutathione; iNOS-inducible nitric oxide synthase; COX—cyclooxygenase; ↑—increased; ↓—decreased.

## Data Availability

No data were generated for this publication.
